# Biosurfactants: A Covid-19 Perspective

**DOI:** 10.3389/fmicb.2020.01341

**Published:** 2020-06-09

**Authors:** Matthew L. Smith, Stefano Gandolfi, Philippa M. Coshall, Pattanathu K. S. M. Rahman

**Affiliations:** ^1^Centre for Enzyme Innovation, School of Biological Science, Institute of Biological and Biomedical Sciences, University of Portsmouth, Portsmouth, United Kingdom; ^2^TeeGene Biotech, Wilton Centre, Redcar and Cleveland, Redcar, United Kingdom

**Keywords:** biosurfactants, Covid-19, handwash, soap, cleaning products, drug delivery

## Abstract

The recent outbreak in severe acute respiratory syndrome – coronavirus-2 (SARS-CoV-2) has demonstrated the complete inability of nations across the world to cope with the pressures of a global pandemic, especially one in which the only current feasible treatments are those which deal with the symptoms alone and not the viral cause. As the death toll rises, scientists begin to fall toward new avenues of research, with novelty showing itself to be an incredible and so far, underrated resource. In this case, the use of biosurfactants in dealing with this pandemic justifies extensive study with their potential applications being in the prevention of viral spread; dealing with the symptoms that develop after the incubation period; directly targeting viral infected cells and preventing the spread of the virus throughout the host, all in addition to also acting as potential drug delivery systems and cleaning agents. This extensive avenue of biosurfactants owes to the simplicity in their amphiphilic structure which permits them to interact directly with the lipid membrane of the coronavirus, in a way which wouldn't be of significant threat to the host. Although it could possibly interact and affect the virus, it could also affect human internal organs/cells by interacting with lipid membrane, if (biosurfactant is) ingested, and it still needs further studies in human models. The structure of the coronavirus, in this case SARS-CoV-2, is detrimentally dependent on the integrity of its lipid membrane which encloses its vital proteins and RNA. Biosurfactants possess the innate ability to threaten this membrane, a result of their own hydrophobic domains across their amphiphilic structure. With biosurfactants additionally being both natural and sustainable, while also possessing a remarkably low cytotoxicity, it is of no doubt that they are going to be of increasing significance in dealing with the current pandemic.

## Introduction

SARS-COV-2 poses a serious and escalating threat to public health, after having triggered a pandemic that stems from its first recorded infection in Wuhan, China during early December 2019 (Hong et al., 2020). In many cases, infection will induce flu-like symptoms in the host who will additionally become highly contagious, having an averaged R_0_ of roughly 3.28, even throughout the initial incubation period which has been suggested to last up to 3–14 days (Backer et al., 2020; Liu et al., 2020). Complications in the immune response or infection occurring in those who already have underlying health conditions, may result in more serious symptoms such as pneumonia or acute respiratory distress syndrome (ARDS) which can often be fatal. This incidence of fatality paired with the ease of viral transfer which often occurs through droplet and aerosol transmission is what makes this particular strain of coronavirus so deadly (Rothan and Byrareddy, 2020).

There is current debate into the significance of the political action of many countries which could have prevented the spread from having such a global impact and whether more could have been done to better prepare healthcare services to deal with the virus. Having been better equipped, hospitals may have likely been able to decrease the overall rates of death from the virus. Regardless of where the responsibility for this pandemic lies, it is clear that we currently rely on international collaboration and extensive scientific prowess to deal with the issue and recover from it in a way which will best reduce the impact it can cause in the future.

One such avenue of investigation is the use of biosurfactants which have proven themselves to be significant in a variety of processes, all of which being crucial in managing the pandemic by dealing with both the virus itself and the symptoms in which it can cause. The structure of the coronavirus, similarly to others of this type, consists of a lipid membrane which encloses its vital proteins and positive sense RNA (Vellingiri et al., 2020). This is an incredibly simple structure but can cause tremendous harm when its membrane fuses with that of an animal cell, allowing its RNA to be synthesized by the host. This will result in the replication of the virus with the use of the host's cellular mechanisms spreading to other cells in the body, causing exponential damage in the process. The lipid membrane, along with embedded spike proteins is crucial in the virus's ability to both maintain its integrity and also pass our cells' phospholipid bilayer which allows it to initiate its mechanism of infection (Das, 2020). The amphiphilic nature of biosurfactants allows them to interact with the hydrophobic domain of the viral membrane with a significant enough affinity to disrupt it, resulting in a breakdown of its structure and therefore disabling it.

Biosurfactants are currently used across a large range of industrial and medical processes and their innate versatility open up their use for a large variety of coronavirus related applications (Randhawa and Rahman, 2014). In dealing with this pandemic, it is crucial that we target the virus at every stage of its transmission and incubation. The use of biosurfactants will therefore be considered in handwashes and cleaning agents to prevent the spread of the virus; targeting and relieving the symptoms after infection; acting as drug delivery systems and additionally their use in other important areas with a key example being the production reliable antiviral facemasks.

## Biosurfactant Structure and Function

Biosurfactants are defined as being amphiphilic moieties which possess the ability to reduce surface tensions across the interface of typically polar substances such as oil and water, therefore exhibiting emulsification properties. Biosurfactants stand out from synthetic surfactants mainly because of their biological and therefore renewable origins, being predominantly made by microbial species and some plants. Compared to their synthetic counterparts biosurfactants have greater emulsification activities, work across a broader range of temperature conditions and most importantly, they have been proven to exhibit a significantly low degree of cytotoxicity (Abdel-Mawgoud et al., 2010). The amphiphilic nature of biosurfactants means that their hydrophobic domain is able to interact with the lipid membrane of the virus, while simultaneously interacting with other hydrophilic substances such as water. This property is what allows them to disrupt the virus structure and therefore deactivate it (Sandeep and Rajasree, 2017).

Biosurfactants additionally have an interesting trait in which they are able to form micellar structures around their critical micelle concentration (CMC), a value that differs greatly between the different biosurfactant types. This structure will be significant in directly targeting the virus, impacting its overall emulsification activity, while also being crucial in our application of biosurfactants in drug delivery. The micelles have the potential to work as liposomes which could directly deliver a drug to the site of infection while also protecting it from the harsh conditions in the body which would otherwise impact its function (Nakanishi et al., 2009).

The versatility in the use of biosurfactants alongside their already large presence in both the pharmacological and food industries prove them to be a significant route in finding novel solutions to the COVID-19 pandemic, therefore justifying their extensive research moving forward (Campos et al., 2013; Fracchia et al., 2018; Nitschke and Silva, 2018; Ribeiro et al., 2020).

### Mechanism of Action of Biosurfactant in Humans

Due to biosurfactant unique chemical structures, understanding the functional mechanisms of action as well as their toxicity to human body is crucial for their exploitation in medical field. Nowadays, biosurfactant find applications as antimicrobial, anti-adhesive, in immunomodulation and as antitumor. Lipopeptides and glycolipids are the most effective as antimicrobial and represents an important source for the discovery of new antibiotics. Biosurfactant have been shown in mammalian cells, to participate in several intercellular molecular recognition steps such as signal transduction, cell differentiation and cell immune response acting as antitumor agents by interfering with cancer progression processes (Gudiña et al., 2013; Fracchia et al., 2015; Sajid et al., 2020).

The antimicrobial and anti-adhesive properties of biosurfactant relays on membrane damage/disruption, causing metabolite leakage by modification of membrane protein morphology; by affecting energy generation and metabolites transport as well as by altering the bacterial lipopolysaccharide system (LPS), thus reducing cell adhesion and biofilm formation (Van Hamme et al., 2006; Fracchia et al., 2015). Different lipopeptides have reached a commercial antibiotic status, like echinocandins, micafungin, anidulafungin, and daptomycin (Fracchia et al., 2015). Moreover, some biosurfactant have shown immunomodulation activities (Sajid et al., 2020). As examples surfactin is an interesting molecule, believed to reduce the activity of macrophage by down regulating the expression of several cell surface molecule (i.e., CD54), thus being a potential candidate in the treatment of hypersensitivity related immune disorders (Paine et al., 2002). Surfactin is also known to have anti-inflammatory activities because of its inhibitory properties on phospholipase A2, on the release of interleukin and nitric oxide (Kim et al., 1998; Byeon et al., 2008; Backhaus et al., 2017). In a similar way sophorolipid injection in animals showed inhibition of pro-inflammatory cytokine and nitric oxide in the treatment of sepsis (Fu et al., 2008). Biosurfactant have been also proposed as new molecule for the treatment of autoimmune diseases as well as potent immuno-modulator and anticancer agent (Sajid et al., 2020).

Despite their versatility some biosurfactant are produced by opportunistic bacteria and it is essential to consider their *in vivo* toxicity and safety. Scarcity of clinical data on the use and validation of such molecules in animal models and human volunteers pose a major challenge. Nonetheless, some biosurfactants have proven their efficacy in different sectors, fulfilling drug regulatory bodies requirements as biocompatible and non-toxic molecules.

### Anti-viral Applications of Biosurfactants

Although reasons behind microbial biosurfactant production are currently unresolved, a likely explanation has been proposed through evolutionary analyses. This view recognizes competitive advantages generated through biosurfactant production, aiding in areas such as resource acquisition and defense, therefore increasing survivability when compared to other organisms who may be disadvantaged as a result (Cameotra et al., 2010; Kiran et al., 2015). Biosurfactant production has often been found to occur where species have experienced depleted resources, as well as during times where they may benefit from their antimicrobial nature. Previous studies have explored the defensive nature of surfactants by expanding the application of bioactive peptides to inactivate enveloped viruses. Cyclosporin A (CsA) is a bio-peptide produced from the fungus *Tolypocladium inflatum*, already known to inhibit the propagation of the influenza virus by interfering with the viral cycle (Garoff et al., 1998; Khan, 2017). CsA does not affect adsorption or RNA replication but instead inhibits steps after protein synthesis, such as assembly or budding (Garoff et al., 1998). This is extremely important as budding enables viruses to exit host cells and attach to derived membranes enriched in viral proteins encouraging spread and infection (Hamamoto et al., 2013). By targeting later events of the virus life cycle the problem of resistance to available drugs will be overcome as well as limiting spread. Lipopeptide used as adjuvant or linked to low mass antigenic molecules have also been used to stimulate the immune system to produce antibodies. Synthetic lipopeptide vaccines have been shown to be able to induce virus specific cytotoxic T-lymphocytes against the influenza nucleoprotein epitope (Deres et al., 1989). Similar results have been observed against foot-and-mouth disease *in vivo* B- and T_h_-cell response to HIV-1 (Wiesmüller et al., 1989; Loleit et al., 1996). This might have a very interesting application in novel vaccines discovery and production. Sophorolipids (SL), a group of microbial glycolipids produced by yeasts have shown properties as immunomodulators, anti-inflammatory and improved sepsis survival in experimental animal models (Borsanyiova et al., 2016). By acetylation of the sophorose head groups, SL have been active against herpes virus and HIV virus. Such modification is considered to improve hydrophilicity of SL thus promoting its antiviral and cytokine-stimulating property (Shah et al., 2005; Gross and Shah, 2007).

These are potential issues that may arise with SARS-CoV-2. This necessitates for the screening of potential agents with novel modes of action to eliminate harmful life-threatening effects.

### Biosurfactants Applied as Cleaning Products

Timely antiviral administration is key throughout global pandemics and the prompt treatment of ill individuals is key to managing future cases. Extreme measures such as closing public areas and social distancing may reduce the infection rate of a disease. However, only antiviral drugs or vaccines are effective in curing and preventing infection when directly exposed to SARS-CoV-2. When facing a new and previously uncategorized virus, vaccine production is likely to be a slow process—unless the viral strain closely resembles a previously identified virus in which we have pre-established treatments. Therefore, during such unprecedented times, before a cure is available it is vital to encourage safe and efficient cleaning procedures that effectively eliminate dormant forms of the virus that may lay on surfaces in public areas, clothes or homes.

**Figure 1:**
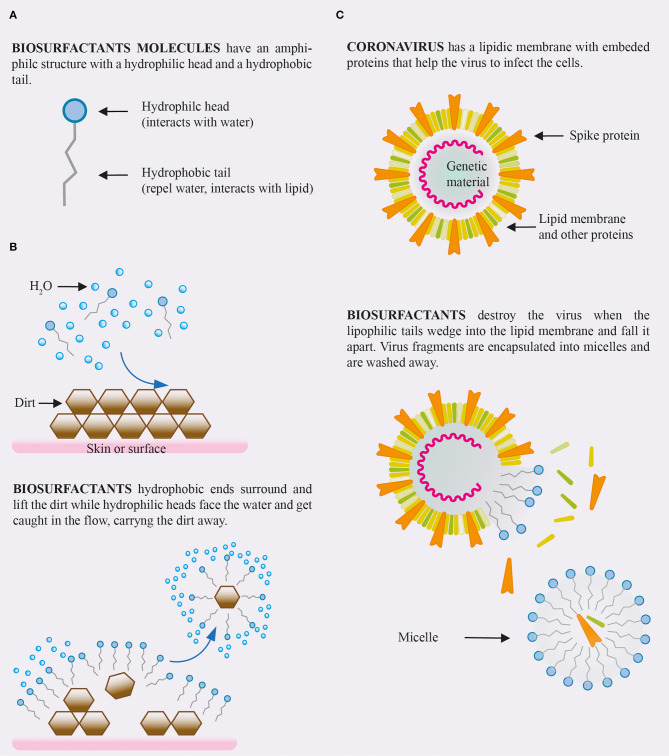
**(A)** Structure of a biosurfactant molecules. **(B)** Interaction of biosurfactants solution with dirt. **(C)** Expected effect of biosurfactants on coronavirus.

Anionic surfactant types are commonly used in cleaning products and detergents. When applied to a surface the fatty acid chains of the surfactant bind to the hydrophobic components of microbes, while the surfactants hydrophilic domain will simultaneously bind to water with a significant affinity, resulting in the solubilization of that microbe. This emulsification reaction therefore enables the surface to be cleaned whilst depositing an effective surfactant layer. Once attached the anionic detergent particles electric charge removes harmful substances from the surface and solubilize them into smaller droplets which results in an emulsification of dirt and detergent. As the surfactant molecules are continuously attached to the dirt/harmful substance the process of repulsion continues effectively preventing the same particle from being reintroduced to the surface. A visual is shown in Figure 1.

Surfactants are the single most important ingredients in laundry and household cleaning products and typically account for 15–40% of total detergent composition (Yangxin et al., 2008). Through increased research, various combinations of surfactant types as well as alterations to surfactant volume within existing and new products have proven surfactants to be effective over traditional products such as bleach. There is no doubt that bleach has proven itself to be an incredible antimicrobial agent with its active ingredient sodium hypochlorite being effective in destroying bacteria, fungi and viruses. However, bleach can irritate skin, airways and mucus membranes, which suggests prolonged exposure to be harmful. Bleach also decomposes under heat and light and reacts easily with other chemicals decreasing its effectiveness. Improper use of bleach such as deviations from recommended dilutions may also affect performance and its overall use is likely to carry with it its own environmental implications which increase in significance when we consider the large-scale washing of public areas to prevent viral spread. All the issues stated above highlight how using bleach alone for disinfection in public areas or areas of high risk such as hospitals/surgeries can involve injury to health-care workers and increase immunosuppression which may lead to increased susceptibility to SARS-CoV-2 especially in areas where exposure to the virus is more likely [World Health Organization (WHO), 2014]. Therefore, using products that contain biosurfactants in conjunction with or as an alternative to heavily chemical cleaners may be more effective in efficient disinfection.

Biosurfactants have great advantages as eco-friendly and less-toxic alternative to synthetic surfactant and to date, glycolipids (sophorolipids, rhamnolipids, and mannosylerythritol lipids) are the most commercialized in cleaning applications by different companies world-wide. Companies like Saraya, Ecover, and Henkel apply SL in their laundry, dishwashing and cleaning products whereas BASF, Evonik, TeeGene and Unilever are commercializing rhamnolipids and lipopeptide biosurfactants based products (Klosowska-Chomiczewska et al., 2011; Fracchia et al., 2014; Randhawa and Rahman, 2014; Singh et al., 2019).

### Handwash Applications of Biosurfactants

Since the start of the pandemic in December 2019, demand for various products -especially gloves, soaps, disinfectants and hand sanitizers has increased dramatically. A huge emphasis has arisen on the necessity of cleanliness, specifically the efficient cleaning of hands after coming into contact with potentially contaminated surfaces both indoors and outdoors. Due to this, governments globally have had to update and define technical and regulatory standards for such products to ensure safe practice and manufacture whilst dealing with the pandemic. Information regarding proper hand washing techniques as well as products content has been published. Specifically, the UK Government has released technical specifications for the production of hand wash and associated legislation of personal protective equipment (PPE) as well as prerequisites before items are sold. Stricter regulations ensure consistency and guaranteed effectiveness of hand washing products which are vital during times of a pandemic (Cabinet Office GOV.UK, 2020)

The effectiveness of hand sanitizers compared to washing hands with soap and water has been of constant debate as of recently. The C.D.C (Center for Disease Control and Prevention) has stated that using soap when washing hands is more effective than hand sanitizer or water alone [Centers for Disease Control and Prevention (CDC), 2019]. This is because surfactants present in soaps lift and remove microbes, harmful substances and dirt from skin. Furthermore, the lather produced from soap as well as effective scrubbing work well to remove contaminants.

The use of alcohol-based hand sanitizer that contain at least 60% alcohol is still recommended if washing hands with soap is not available. Hand sanitizers do not get rid of all types of germs and may not be as effective where hands are excessively greasy or dirty [Centers for Disease Control and Prevention (CDC), 2019]. Alcohol is extremely effective, but efficacy differs among different types. For example, ethyl alcohol (70%) is a powerful germicide and is considered generally superior to isopropyl alcohol. It is also important to consider the prolonged and repeated use of alcohol-based products such as a disinfectant which can cause discoloration and damage to the skin [World Health Organization (WHO), 2014]. Whereas, TeeGene has reported about lipopeptide and rhamnolipid biosurfactant based cosmetics (Randhawa and Rahman, 2014; Focus on Surfactants, 2015) and Evonik has reported their sophorolipid biosurfactant for skin conditioning, refatting and moisturizers properties to be used in shampoo, shower gel and household cleaners (Focus on Surfactants, 2016) and it also has a potential use in handwash applications.

### Biosurfactants Application in Acute Respiratory Distress Syndrome (ARDS)

Acute respiratory distress syndrome (ARDS) is a progressive medical syndrome, characterized by a build-up of fluid in the patient's alveoli which result in inefficient oxygen transfer across these alveolar membranes into the blood (Matthay et al., 2019). ARDS is often a consequence of an already serious medical concern, in this case Covid-19, with the resulting lack of oxygen to organs contributing to the high fatality rates seen in those who begin to develop symptoms (Ware and Matthay, 2000).

One leading cause for this alveolar fluid build-up, in response to infection by SARS-CoV-2, is surfactant dysfunction which has negative consequences on the emulsification and thereby clearance of liquid from this particular region. Current treatments for this rely on ventilators to supplement the body with the oxygen which otherwise would not be able to successfully transfer into the blood, relieving the immediate symptoms which would otherwise lead to issues such as hypoxia (Luks et al., 2020). Socioeconomic factors have played a large role in the effectiveness of such a treatment, with providing enough ventilators and facilities for each patient affected proving to be difficult and, in some cases, impossible considering the cost, space and training required to use them.

With this in mind, biosurfactants present themselves to be a promising area of study in identifying novel treatments for ARDS which could overcome the socioeconomic barriers that have so far limited the effectiveness of ventilators. It is for this reason that future study for their use as a direct treatment for ARDS, through solubilizing the alveolar substrate, is likely to generate positive results and is therefore crucial in combating Covid-19 in the future.

### Biosurfactants and Drug Delivery Mechanisms

When considering possible treatment opportunities for the COVID-19 pandemic, it is crucial to decide upon a mode of drug delivery which doesn't compromise the molecular nature of the product while also being able to deliver it successfully to the area of interest. With the SARS-CoV-2 predominantly impacting the respiratory system and upper gastrointestinal tract, a likely mode of delivery would be via aerosol or lozenge. The micellar nature of biosurfactants result in them being the ideal candidates for either system of drug delivery, allowing them to form a stable liposome which will encase the drug, protecting it from damage which may otherwise cause dysfunction (Sosnowski and Gradon, 2009). The physicochemical characteristics of biosurfactants allow them to maintain their integrity while used in an aerosol, this would be the likely mode of drug delivery considering the main area of virulence to be within the lungs.

The solubility of biosurfactants will work to their advantage throughout this process as they will evidently increase the bioavailability of the drug, once it has been administered. This self-solubilising nature of biosurfactants therefore advances the drugs dose proportionality, resulting in more consistent impacts across patients (Omkar et al., 2020). The ability for biosurfactants to mediate drug delivery is apparent, however the benefits of their use in this way are 2-fold. In addition to providing safe passage for the drug to the target, the biosurfactants will also exhibit natural antiviral properties at the site of infection, in addition to also relieving surfactant dysfunction in the alveoli, another consequence of SARS-CoV-2 infection. In this way, they will be able to inhibit a number of viruses present around that given area while also directly relieving symptoms, a significant factor reducing its virulence and transmission between hosts. This characteristic has furthermore been expanded upon as researchers ponder the ability for biosurfactants to themselves behave as a treatment in this way. One such example includes the addition of clinically approved biosurfactants in gummies or lozenges, as they are consumed the biosurfactants will directly reach parts of the mouth and esophagus which may be impacted and therefore provide symptomatic relief (Vellingiri et al., 2020). In addition to this, the biosurfactant will likely form a vapor in the mouth which can be inhaled through the process of ingestion, allowing it to reach areas of the respiratory tract to potentially provide relief in that area as well.

## Conclusion

The COVID-19 pandemic is one which has had a detrimental impact on public health, seriously hindering the normal functionality of our society and therefore resulting in massive hardship across both our economy and public well-being. It is for this reason that scientists have been at a bid to find novel ways in which this pandemic can be combatted, across all of its planes of influence throughout our society.

Biosurfactants have not only proven themselves to be the ideal candidates to behave as this novel solution but have done so in a way which targets many of the avenues that are crucial in resolving a pandemic of this scale. Their potential in behaving as safe and effective cleaning solutions have been discussed and they have been recognized as being a valid alternative to current procedure. In addition to this, biosurfactants have been found to act as significant drug delivery systems, housing multiple benefits to their use, most importantly showing a great potential in being able to successfully deliver drugs, maintaining dose proportionality in the process. Finally, the potential for biosurfactants to exhibit medicinal qualities in the treatment of SARS-CoV-2 infection, particularly in the relief of symptoms associated with ARDS, have shown exceptional promise. The lack of knowledge shrouding this phenomenon therefore justify extensive future research. A prominent barrier remains in the large production costs associated with biosurfactant bioprocessing, a significant factor which must not be overlooked and should remain a focal point for future study. Reducing the cost of bioprocessing, alongside the continued research of biosurfactant applications in this area are key to success within this field.

The incredible versatility found across the structure and functions of biosurfactants mean that their reach in effectiveness is by no means limited to the applications which have been so far highlighted. As research progresses in this area, the feasibility of both their use and their socioeconomic development increase. The results of this pandemic have been so far detrimental however, it is crucial that we push forward and adapt such ideas in finding the very solutions that we need to combat the consequences of this virus and pathogens in the future. In this way, biosurfactants bring promise to a scenario that is often shrouded in despair, with research and scientific prowess we will not only overcome the issues of this pandemic, but we will additionally better equip ourselves for the future.

## Data Availability Statement

All datasets presented in this study are included in the article/ supplementary material.

## Author Contributions

MS, SG, PC, and PR have equally contributed in the preparation of this manuscript.

## Conflict of Interest

The authors declare that the research was conducted in the absence of any commercial or financial relationships that could be construed as a potential conflict of interest.
